# Contact and Tribological Study of Micro/Nano Groove Texture on the Surface of Gas Bearing Materials Based on Nanoscale

**DOI:** 10.3390/nano13010152

**Published:** 2022-12-28

**Authors:** Liguang Yang, Wensuo Ma, Fei Gao, Shiping Xi, Zhenyu Ma, Zhenhao Ma

**Affiliations:** 1 School of Mechatronics Engineering, Henan University of Science and Technology, Luoyang 471023, China; 2 School of Mechanical Engineering, Tsinghua University, Beijing 100084, China; 3 Luoyang Bearing Research Institute Co., Ltd., Luoyang 471039, China

**Keywords:** contact, tribological, texture, gas bearing, nanoscale

## Abstract

As a kind of sliding bearing, the gas bearing is widely used in high-speed rotating machinery. It realizes energy cleaning in the field of high-speed rotating machinery. In order to solve the problem of reducing the service life of gas bearings due to friction during startup and shutdown, we use micromachining technology to process groove textures with different groove widths on the surface of 0Cr17Ni7Al, a common material for gas bearings. A ball–disc friction contrast test is conducted under dry friction conditions with and without texture. The experiment shows that the lowest average friction coefficient of 0.8 mm texture is σ = 0.745. When the friction radius is 22.5 mm, the wear rate of 1.0 mm texture is the lowest at ω = 3.118 × $10^{-4}mm^{3}/N\cdot mm$. However, the maximum friction coefficient reached is σ = 0.898. Under the nanometer scale, the contact between friction pairs is fully analyzed. The influence mechanism of different groove widths, friction impacts and climbing heights on the friction and wear properties of the micromechanical groove texture on the surface of 0Cr17Ni7Al stainless steel is studied at the nano-fractal scale. The effects of different width grooves on the surface texture and tribological properties of the micromachine are studied.

## 1. Introduction

As a kind of sliding bearing, the gas bearing works on the mechanism of gas dynamic lubrication. Compared with traditional bearings, it has the advantages of good impact resistance, a wide temperature range, a self-adaptive nature, cleanliness, a long service life and high speed [1,2]. It is widely used in high-speed rotating machinery. The appearance of the gas bearing has completely eliminated the disadvantage of environmental pollution caused by leakage of traditional bearing oil circuits. When the bearing is used, it is connected with the atmosphere, and no additional air source is required. It really realizes energy cleaning in the field of high-speed rotating machinery. Many scholars have studied the measurement of gas flow and the viscoelasticity of fluid flow [3,4]. However, contact friction between the low-speed rotor and gas bearing during startup and shutdown is the main factor that affects the service life of the gas bearing. This kind of contact friction will cause problems such as surface wear and service life reduction. Therefore, it is extremely important to solve the friction problem in the process of starting and stopping for the reliability of the gas bearing.

Studies performed by a large number of scholars have shown that adding lubricant [5,6,7,8,9,10,11] to the object surface or using micro/nano texture formation to modify the friction pair surface can effectively improve the tribological properties of the material surface [12,13,14,15,16,17]. This surface modification method is called surface texture, which is used to prepare a certain size and geometric shape on the friction pair surface using micro/nano-machining technology. It has a good antifriction effect. Surface texture is widely used in the hydrophobic [18,19], antifriction [20,21,22], friction-increasing [23] and wear-resistant [24,25] fields. At present, there are many methods to prepare surface texture, mainly micro/nano-scale processing, but including ultrasonic processing technology, ion etching [26,27], electrochemical processing technology [28,29], chemical etching [30,31,32], laser processing [33,34,35,36] and micromachining technology [37,38].

Inspired by the above literature, our research team used micromachining technology to process groove textures with different groove widths on the surface of 0Cr17Ni7Al, a common material for gas bearings. A ball–disc friction contrast test was conducted under dry friction conditions with and without texture. In the nanometer scale, the contact between the friction pairs was analyzed macroscopically and microscopically. The surface wear morphology and friction wear state of the samples were analyzed by means of a three-dimensional white light interferometer, scanning electron microscope (SEM) and energy-dispersive spectrometer (EDS). The influence of grooves with different widths on the tribological properties of textured surfaces was studied. The friction and wear behavior and the antifriction mechanism of the micromachined surface texture in the wear process were analyzed and discussed.

## 2. Contact Analysis

To ensure the single variability of the experiment, all the texture samples have groove width variation only, and the other parameters are consistent. The parameter settings of the texture samples are shown in Table 1.

Next, we will analyze the contact condition of the friction pair.

### 2.1. Macro Contact of Friction Pair

**Figure 1:**
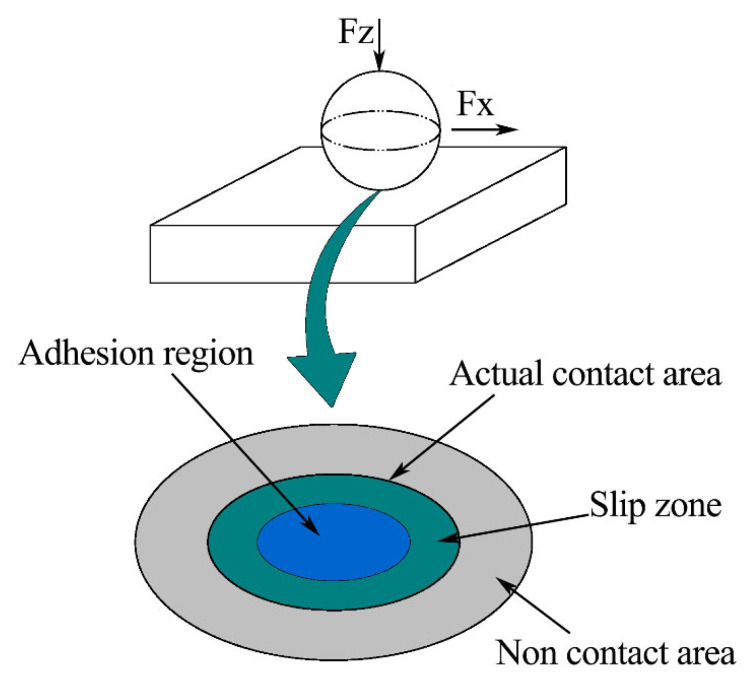
Macro contact.

At the beginning of the experiment, the macro contact between the ball and the disc conforms to Hertz’s theory [39], as shown in Figure 1.

From Figure 1, we can see an elliptical contact area. Moreover, the size of the contact area is much smaller than the size of the object.

### 2.2. Micro Contact of Friction Pair

When the experiment is carried out for a certain period of time, the surface of the friction pair becomes worn, and the surface of the ball gradually becomes flat. At this time, the friction pair is in the contact stage of two rough surfaces. The contact pattern has changed. We use the statistical contact model to view the contact situation from a microscopic perspective.

#### 2.2.1. Micro-Convex Body Contact

**Figure 2:**
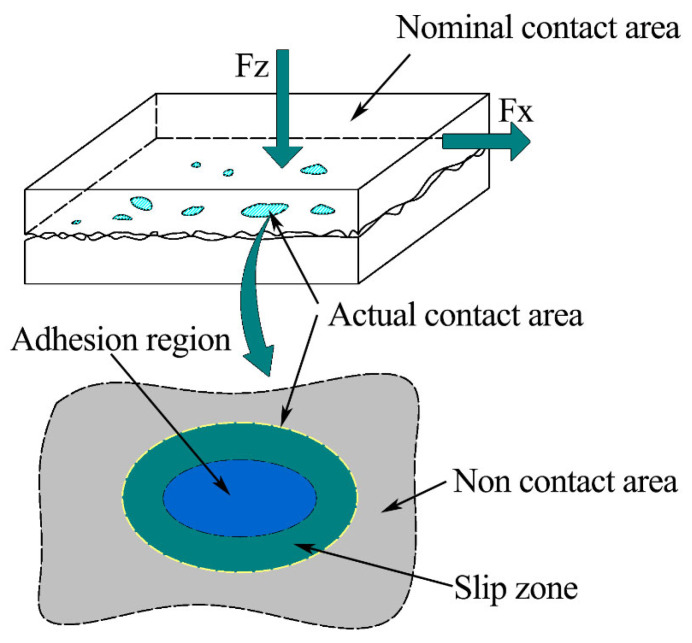
Actual contact diagram of micro-convex body.

The existence of roughness means that only small parts of the two objects come into contact and bear normal load. The statistical contact model is shown in Figure 2. Even for the part that is actually in contact, due to the difference of normal deformation of the rough peak and the influence of plastic deformation, the normal load actually borne by each rough peak is different. We use the statistical model to study the plane contact problem. The rough peaks are regarded as a series of micro-convex bodies with statistical parameters covering the absolutely smooth surface. Each micro-convex body is independent of the others. The final contact effect is the sum of multiple single peak contact effects.

The GW statistical contact model proposed by Greenwood and Williamson assumes that the height and the radius of curvature of the micro-convex body are parameters that can be described via a probability density function [40]. According to the calculation method given by McCool [41], the surface roughness parameters are as follows:

(1) $$\begin{array}{l} m_{0}=E\left[z^{2}\right]\\ m_{2}=E\left[{\left(\frac{dz}{dx}\right)}^{2}\right]\\ m_{4}=E\left[{\left(d^{2}z/dx^{2}\right)}^{2}\right] \end{array}$$

(2) $$\{\begin{array}{l} R=0.375\sqrt{\pi /m_{4}}\\ \eta =m_{4}/6\pi \sqrt{3}m_{2}\\ \sigma =\sqrt{m_{0}} \end{array}$$

Formula (2) is derived from Formula (1), where *E* is expectation. Generally, *R*, *η* and *σ* are used to calculate the statistical model. In addition, the height distribution function of asperities and material properties are also important parameters. The relationship between the surface profile and the root mean square of the micro-convex body height can be expressed using the same formula as the Gaussian distribution [42]. The formula is:

(3) $$\sigma_{a}=\sqrt{m_{0}\cdot }\sqrt{1-0.8968/\alpha }$$

where $\alpha =\left(m_{0}m_{4}\right)/m_{2}^{2}$. The GW model is based on the Hertz contact theory of a micro-convex body. The real contact area *A_r_* and normal load *W* are calculated via a statistical method as follows:

(4) $$\{\begin{array}{l} A_{r}=\pi \eta A_{n}R\int_{d}^{+\infty }\left(z-d\right)\varphi \left(z\right)dz\\ W=\frac{2}{3}\eta A_{n}ER^{1/2}\int_{d}^{+\infty }{\left(z-d\right)}^{3/2}\varphi \left(z\right)dz \end{array}$$

Here, *φ*(*z*) is the distribution function of the rough peak height.

It can be seen from Formula (3) that the GW model requires three parameters: the height distribution, the curvature radius and the distribution density of the micro-convex body. These parameters will be difficult to measure [43]. It can be seen that both the macro-contact and micro-contact model analyses conform to the Hertz contact theory.

#### 2.2.2. Fractal Contact at Nanoscale

**Figure 3:**
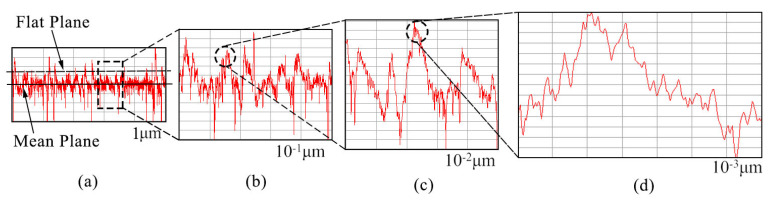
Schematic diagram of contact between nanoscale fractal surface and rigid rough plane: (**a**) Rigid contact surface; (**b**) Schematic diagram of rigid contact surface after being magnified by 10 times; (**c**) Schematic diagram of rigid contact surface after being magnified by 100 times; (**d**) Schematic diagram of rigid contact surface after being magnified by 1000 times.

Statistical contact models need to measure the surface profile in advance and give the height distribution density function. There is a problem with the sampling interval or instrument measurement resolution. To overcome this problem, Majumdar and Bhushan [44] introduced fractal theory to describe the surface profile. One of the advantages of fractal theory is that the contour depicted has cross-scale self-similarity, which is independent of the measurement scale. It is found that the length of some geometric shapes will change with the change of measurement scale, but the fractal dimension has scale invariance. This means that the surface profile is statistically self-affine. That is, when the surface profile is magnified, it can be found that the magnified profile is similar to the original profile. Figure 3 shows the enlarged schematic diagram of the nanoscale one-dimensional fractal surface profile. Figure 3a shows the contact between the rough surface with fractal characteristics and the rigid rough surface. The morphology shown in Figure 3b can be obtained after the local part is magnified by 10 times. After the local morphology in Figure 3b is further magnified by 10 times, the contour morphology shown in Figure 3c can be obtained. After the local morphology in Figure 3c is further magnified by 10 times, the contour morphology shown in Figure 3d can be obtained.

Therefore, the roughness surface morphology changes from micrometer level to nanometer level through three times’ amplification of the fractal. We can find that the fractal surfaces have self-similar properties. After the contact analysis, we continued the experiment.

## 3. Materials and Methods

### 3.1. Micromachining Plate

Micromachining is a machining method based on a micro/nanoscale. This method has the advantages of controllable size, no material limitation and low cost. It is widely used to prepare groove textures.

**Figure 4:**
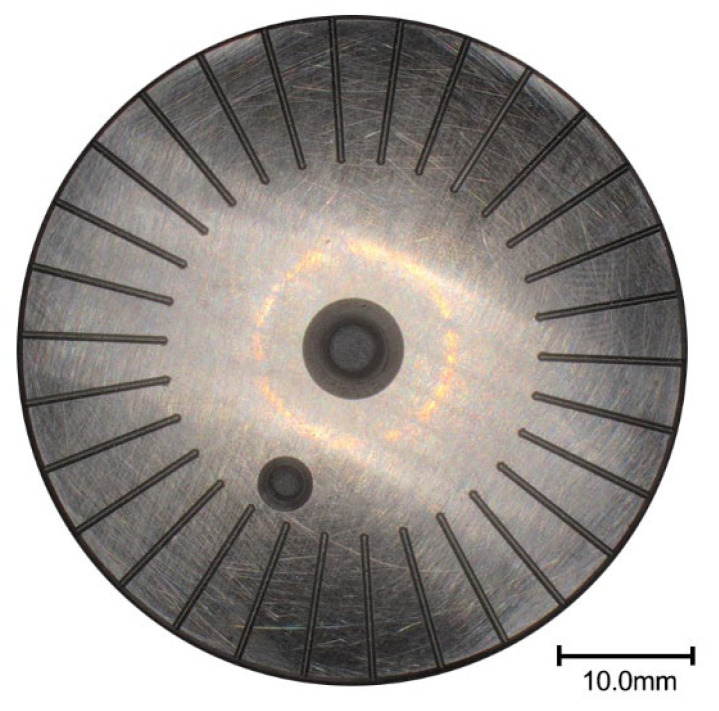
Surface texture disc test piece.

The test samples in this paper were processed and prepared using the JDGR200T high-speed machining center produced by Beijing Jingdiao Technology Group Co., Ltd. (Beijing, China). The sample was processed using a ball-end milling cutter. The main process parameters of the machining center equipment are shown in Table 2. The surface texture preparation process for disk test pieces is as follows: raw materials (bars) → mechanical processing (turning, grinding, heat treatment, hole grinding, and flat grinding) → sample texture preparation (polishing, cleaning, and drying) → micromechanical equipment debugging (processing methods and path parameters) → micromechanical texturing (shape, size, and spacing) → sample post-processing (cleaning and drying). The final surface texture disc specimen is shown in Figure 4. The chemical composition and mass fraction of the friction pair are shown in Table 3. We also mentioned this in previous studies [22].

### 3.2. Friction and Wear Test Material

The friction and wear properties of the groove texture on the 0Cr17Ni7Al surface were studied using the MFT-5000 tester. The friction and wear test adopts the test method of fixing the upper test piece (ball) and rotating the lower test piece (disc). 

Friction tests were carried out on five specimens. Each group of samples was further ultrasonically cleaned with acetone before testing, and the surface of the test piece was wiped with ether. After the test, a white light interferometer, scanning electron microscope and energy dispersive spectrometer were used to detect the wear mark on the surface of the sample. The change of friction coefficient was analyzed.

### 3.3. Friction and Wear Calculation

The specific experimental parameters are shown in Table 4. A white light interferometer was used to detect the cross-section profile of the wear trace. The wear sectional area is calculated using the profile integral. The amount of wear is obtained by multiplying the total wear distance by the wear area [36]:

(5) $$w=\frac{V}{F\cdot S}$$

where *w* is the wear rate, 10^−4^mm^3^/N·mm; *V* is the wear volume, mm^3^; F is the normal load, N; and *S* is the running distance, mm. In this study, the error is reduced by the average value of the wear rate of three parallel tests.

## 4. Results and Discussion

### 4.1. Friction Coefficient and Wear Rate

**Figure 5:**
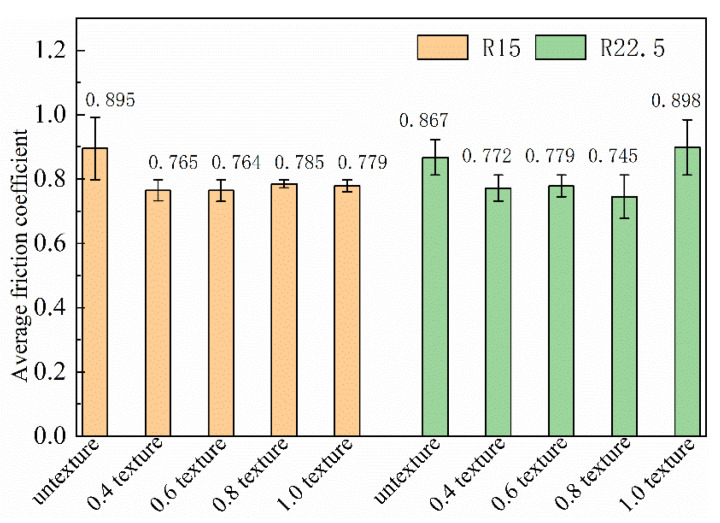
Friction coefficient error band of different samples.

For the convenience of understanding the research content, we summarize the experimental data such as the friction coefficient and wear rate of all the samples in this experiment in Table 4. It can be seen from Table 4 and Figure 5 that, at the turning radius of 15 mm, the friction coefficient without texture is the largest, and the friction coefficient is relatively dispersed as a whole. This shows that the friction coefficient of the untextured surface is unstable. The friction coefficients of the 0.4 mm, 0.6 mm, 0.8 mm and 1.0 mm textures are lower than those of the samples without textures. It can be inferred that surface texture has a good antifriction effect. Moreover, the friction coefficient is relatively concentrated and stable. We also mentioned this stationarity in previous studies [22,36]. The change trend of the friction coefficient for the five groups of friction experiments under a 15 mm rotating radius is untextured > 0.8 mm texture > 1.0 mm texture > 0.4 mm texture > 0.6 mm texture. We can also find that the friction coefficient of the 1.0 mm texture fluctuates greatly and exceeds the surface without texture when the rotation radius is 22.5 mm. The friction coefficients of the 0.4 mm, 0.6 mm and 0.8 mm textured surfaces are relatively stable and significantly lower than those of the untextured surfaces. The friction coefficient of the five groups of friction experiments under a 22.5 mm rotating radius is 1.0 mm texture > untextured > 0.6 mm texture > 0.8 mm texture > 0.4 mm texture. This shows that not all textures with a groove width have good antifriction performance at any radius of rotation.

Compared with the untextured surface, the groove texture causes contact discontinuity between the ball and the disc and may be the main reason for friction reduction of the textured surface. The accumulation of wear debris in the sliding direction is reduced, the adhesion tendency is weakened, and the wear resistance is improved. However, at the same time, groove texture is bound to increase the contact stress. When the negative effect of contact stress is greater than the positive effect mentioned above, the friction coefficient of the 1.0 mm texture at 22.5 mm will increase.

**Figure 6:**
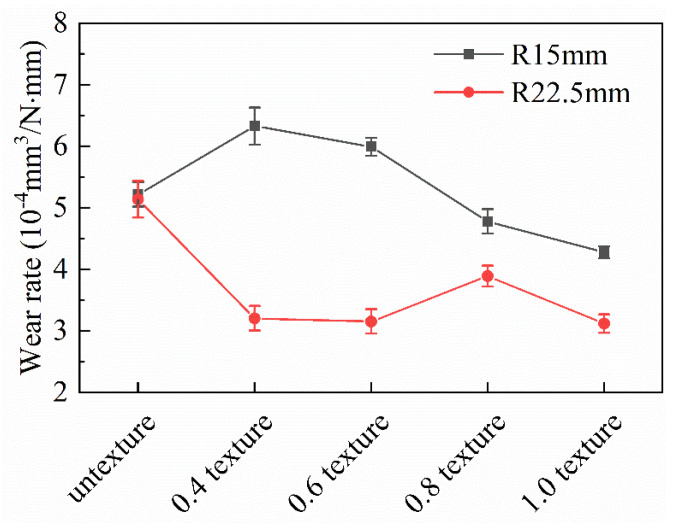
Wear rate of different samples.

Table 4 and Figure 6 show the trend of wear rate on a broken line graph under a 15 mm and 22.5 mm rotating radius. The wear rates of the 0.4 mm texture and 0.6 mm texture exceed that for the surface without texture. However, the wear rates of the 0.8 mm texture and 1.0 mm texture are relatively stable and significantly lower than those of untextured surfaces. The wear rates of the five groups of friction tests under a 15 mm rotating radius are 0.4 mm texture > 0.6 mm texture > untextured > 0.8 mm texture > 1.0 mm texture. All textures under the 22.5 mm rotating radius are more wear-resistant than untextured surfaces, and the wear resistance is obvious. The wear rates of the five groups of friction tests are untextured > 0.8 mm texture > 0.4 mm texture > 0.6 mm texture > 1.0 mm texture. It can be seen that the wear rate of the untextured surfaces is basically the same no matter whether using the 15 mm or 22.5 mm rotating radius, and the wear is relatively severe. However, the 0.4 mm texture and 0.6 mm texture show great dispersion. The wear rates of the 0.8 mm texture and 1.0 mm texture are close and obviously lower than those of the untextured surfaces. Except for the untextured surface, the wear rates of the four groups of texture generally decreased with an increase in texture width, and the regularity is obvious. From the above analysis, it can be found that not all textures have good wear resistance.

According to Table 4 and Table 5, the total number of collisions of the 0.8 mm groove texture at the 22.5 mm friction radius reached 24,000, and the total climbing height is 408 mm. The minimum average friction coefficient is σ = 0.745. The lowest wear rate can be seen for the 1.0 mm texture with a friction radius of 22.5 mm, and the lowest wear rate is ω = 3.118 × $10^{-4}mm^{3}/N\cdot mm$. At this time, the total number of collisions is 24,000, and the total climbing height is 499.2 mm, but the maximum friction coefficient reached is σ = 0.898.

**Figure 7:**
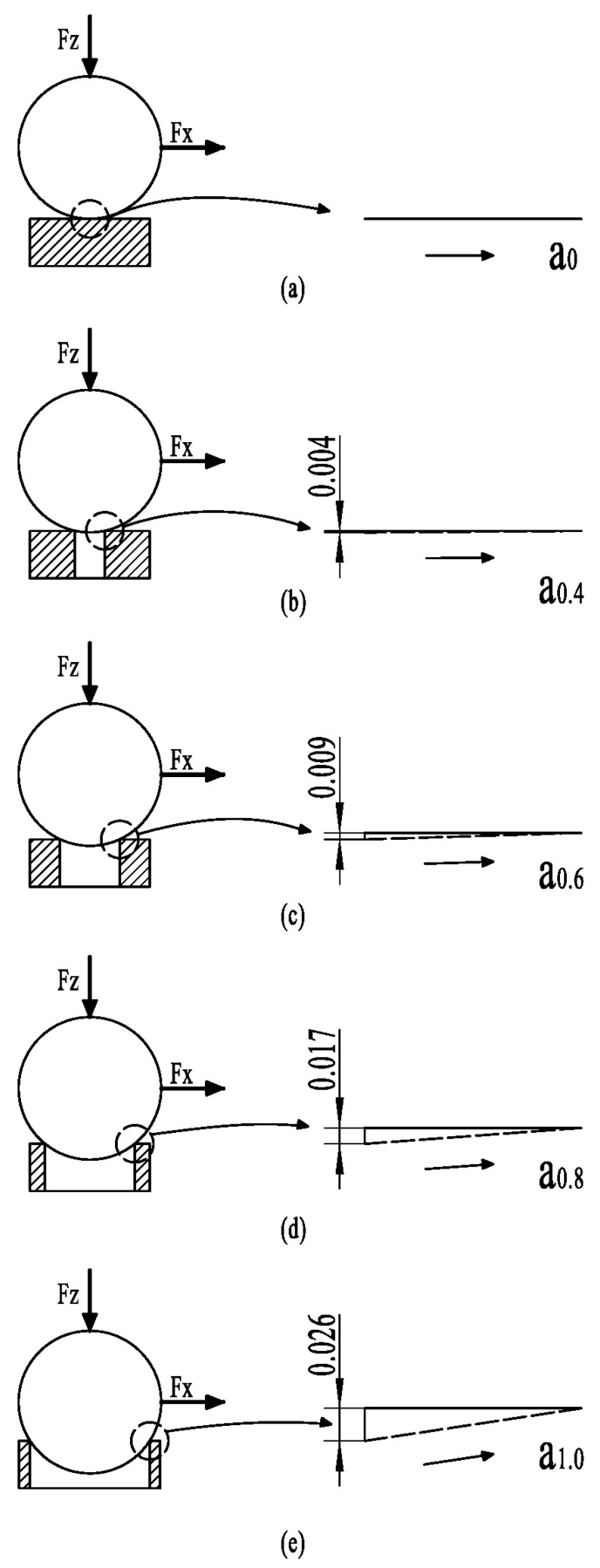
Schematic diagram of collision climbing: (**a**) untexture; (**b**) 0.4 mm groove width mechanical texture; (**c**) 0.6 mm groove width mechanical texture; (**d**) 0.8 mm groove width mechanical texture; (**e**) 1.0 mm groove width mechanical texture.

From the friction-reducing and wear-resistance mechanisms of a conventional groove texture, the reason the groove-textured surface has better friction reduction and wear resistance than the untextured surface may be that the wear debris on the groove-textured surface is removed with friction during the friction process. A part of the wear debris from the groove texture is captured and stored, thus reducing the further aggravation of abrasive wear. Some of the debris collides and rolls. Under the same test parameters, the surface friction without texture gradually increases with the friction test. This is mainly because the wear debris cannot be discharged in time during the friction process. The generated wear debris forms three-body wear on the surface of the friction pair. The heat generated by friction is increased so that the wear is constantly aggravated. This is a conventional wear mechanism, but it is not comprehensive. Next, we will further analyze the friction mechanism of the groove texture. In fact, in the actual friction process of the groove texture, there are different contact friction stages. According to the order of friction time, we can divide it into the first contact friction stage and the second contact friction stage. In the first contact friction stage, the contact of the friction pair conforms to the macroscopic Hertz contact in Figure 1. At the same time, friction collision and friction heat generation are coupled. In order to facilitate the reader’s understanding, we introduce the concepts of climbing and climbing height. Climbing refers to the slope that the ball slides along from the groove to the disc surface in the sliding direction during friction. The climbing height is the vertical height of the ball from the deepest position of the groove to the surface of the disc. We consider that the untextured surface is a groove texture with a groove diameter of 0 mm. So, this research is equivalent to the experimental comparison of five groove textures with different groove widths under the same friction experimental parameters. During the initial friction between the ball and groove texture, the climbing contact diagram of a ball with a diameter of 9.525 mm and a surface texture with groove widths of 0 mm (untextured), 0.4 mm, 0.6 mm, 0.8 mm and 1.0 mm, respectively, is shown in Figure 7. The moving tracks of the contact points of the five width textures in the climbing process are respectively represented by $a_{0},\;a_{0.4},\;a_{0.6},a_{0.8},\;\mathrm{and}\;a_{1.0}$. It can be seen that, with an increase in texture width, the climbing height gradually increases.

Theoretically, the horizontal distance of the climb is the same. At this time, the higher the climbing height, the longer the climbing distance. Therefore, the more severe the wear is, the greater the friction coefficient will become. Due to the need for different groove widths, the diameter of the upper test piece (ball) ground into a plane is larger and takes longer to reach face-to-face friction. Therefore, the higher the climbing height, the more severe the wear. It seems straightforward to use this as a measure of the tribological properties of grooved textured surfaces.

However, that is not the case. As shown in Table 5, with the change of the friction radius, under the same sliding linear velocity, time and load, the frequency of collision between the upper specimen and the groove on the surface of the lower specimen during friction is different. In addition, when the ball is in contact with the groove, friction and collision occur constantly, which makes the friction pair vibrate. Thus, the wear debris will roll and slip, reducing friction. Balls with different groove widths have different climbing heights. The greater the climb height, the greater the forward resistance to overcome. At the same horizontal rotation speed set in the experiment, the same climbing time is required at the same horizontal distance. The dynamic load to overcome climbing resistance increases with an increase in climbing height. Moreover, due to the different climbing heights, the collision and vibration of friction pairs before climbing are also different. The friction and collision heat generated during collision and climbing are also different. In the friction process, textured grooves can reduce the contact area and contact time of friction pairs. Therefore, the increase in friction heat is reduced and achieves the effect of heat dissipation. This effectively slows down the formation of oxidation wear and fretting wear. Therefore, we can simply take climbing height as the decisive factor to measure tribological performance.

In conclusion, different tribological characteristics of different groove widths need to be considered in combination with the coupling effects of nanoscale contact, ball groove collision vibration and the ball climbing effect described in the previous section.

### 4.2. Analysis of Wear Morphology

The coupling effects of friction collision, friction heat generation, friction climbing, etc., have been experienced in the first contact friction stage. The friction contact part of the ball has become a relative plane. This indicates that friction has started to enter the second contact friction stage, and the friction form of this stage has become plane-to- plane contact friction. We will use the aforementioned micro-contact in Figure 2 and the Figure 3 nanoscale micro-convex contact for analysis. The wear mark morphology we are going to analyze in this section mainly comes from the friction process in the second contact friction stage.

The maximum wear-mark depth values are shown in Table 6 and Table 7.

**Figure 8:**
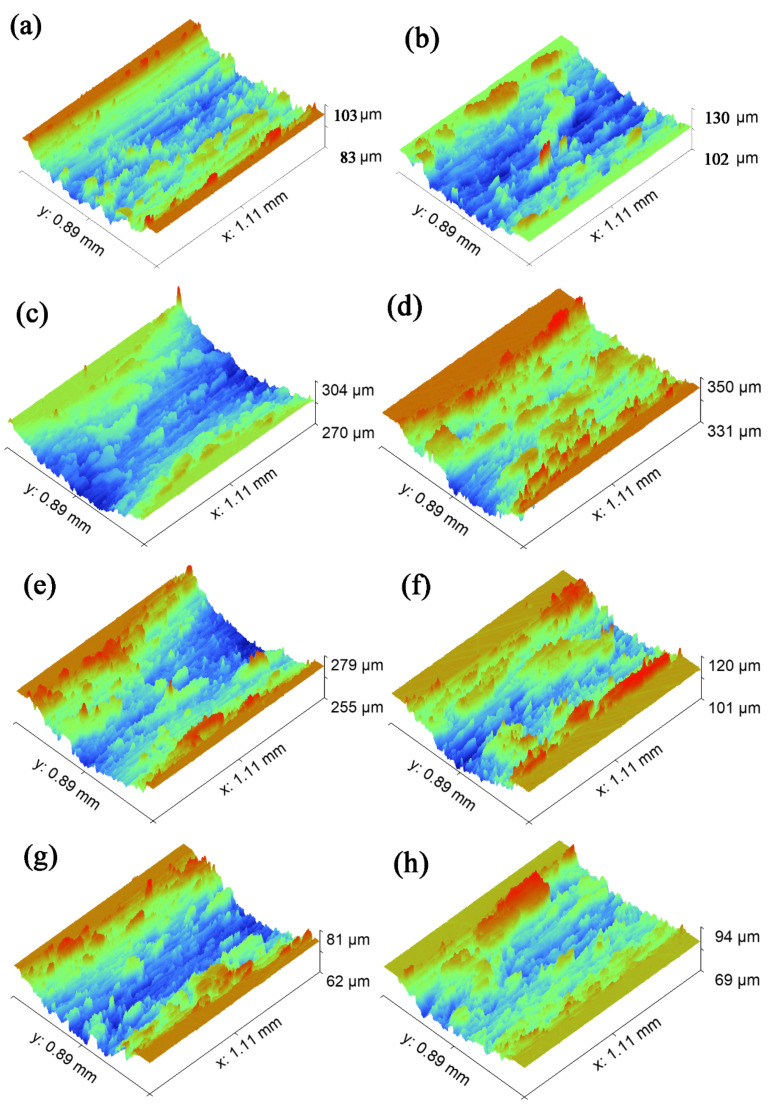

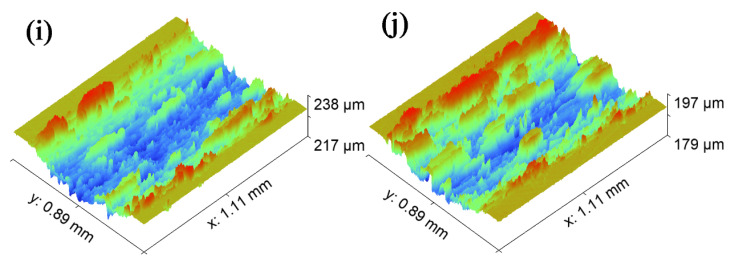
Morphology of wear marks of different samples: (**a**) untextured rotation radius of 15 mm; (**b**) untextured rotation radius of 22.5 mm; (**c**) 0.4 mm groove width mechanical texture rotation radius of 15 mm; (**d**) 0.4 mm groove width mechanical texture rotation radius of 22.5 mm; (**e**) 0.6 mm groove width mechanical texture rotation radius of 15 mm; (**f**) 0.6 mm groove width mechanical texture rotation radius of 22.5 mm; (**g**) 0.8 mm groove width mechanical texture rotation radius of 15 mm; (**h**) 0.8 mm groove width mechanical texture rotation radius of 22.5 mm; (**i**) 1.0 mm groove width mechanical texture rotation radius of 15 mm; (**j**) 1.0 mm groove width mechanical texture rotation radius of 22.5 mm.

It can be seen from Figure 8 that there are different degrees of bulges and flakiness in the middle and on both sides of the wear mark. It was found from previous studies that this sheet is called a hard phase peak [38]. In the second contact friction stage, the asperities of the friction pair migrate with wear debris in the first contact friction process under the combined actions of load and sliding speed. In the subsequent friction process, the hard phase peak is formed due to the agglutination of friction heat and the rapid reduction of temperature, and the agglutination “cold welding” accumulation occurs. According to the position of the wear mark where the hard phase peak is located, it can be divided into an edge hard phase peak and a wear-mark hard phase peak. When the hard phase peak is formed on the surface of the ball and disc, we can see it as a magnified fractal of the micro convex from the micro to the macro. At this time, the hard phase peak is larger in volume and higher in hardness. A hard phase peak is formed on the surface of the friction pair. The edge hard phase peak can form a support on the friction pair surface to protect the surface and slow down further wear. The hard phase peak of the wear mark exists inside the wear mark, which will magnify the effect of the hard peak, further aggravating the plough groove effect on the plate. It even plays a key role in the evolution from abrasive wear to adhesive wear. It can be seen from Figure 8a,b that there is a deep and large plough groove in the wear mark. This is mainly caused by the hard peak of the hard phase peak of the wear mark. Figure 8c also reflects a larger and deeper plough groove, even more severe than Figure 8a,b. As mentioned above, combined with the analysis in Table 5, the 0.4 mm texture in the first contact friction stage has no prominent climbing height, but the number of collisions in the whole friction stage is up to 72,000. Under constant friction, collision, heating and rapid cooling with high frequency, the wear debris is glued by friction, collision and high temperature before it escapes. It then cools rapidly and circulates repeatedly. As a result, the hard phase peak of the wear mark is larger than that of the untextured surface. Figure 8d–j show that most of them are edge hard phase peaks. Combined with Table 4, it can be further confirmed that the edge hard phase peak has an obvious supporting and protective effect on the wear mark.

### 4.3. Electron Microscope and Energy Dispersive Spectrum (EDS) Analysis of Worn Surface

**Figure 9:**
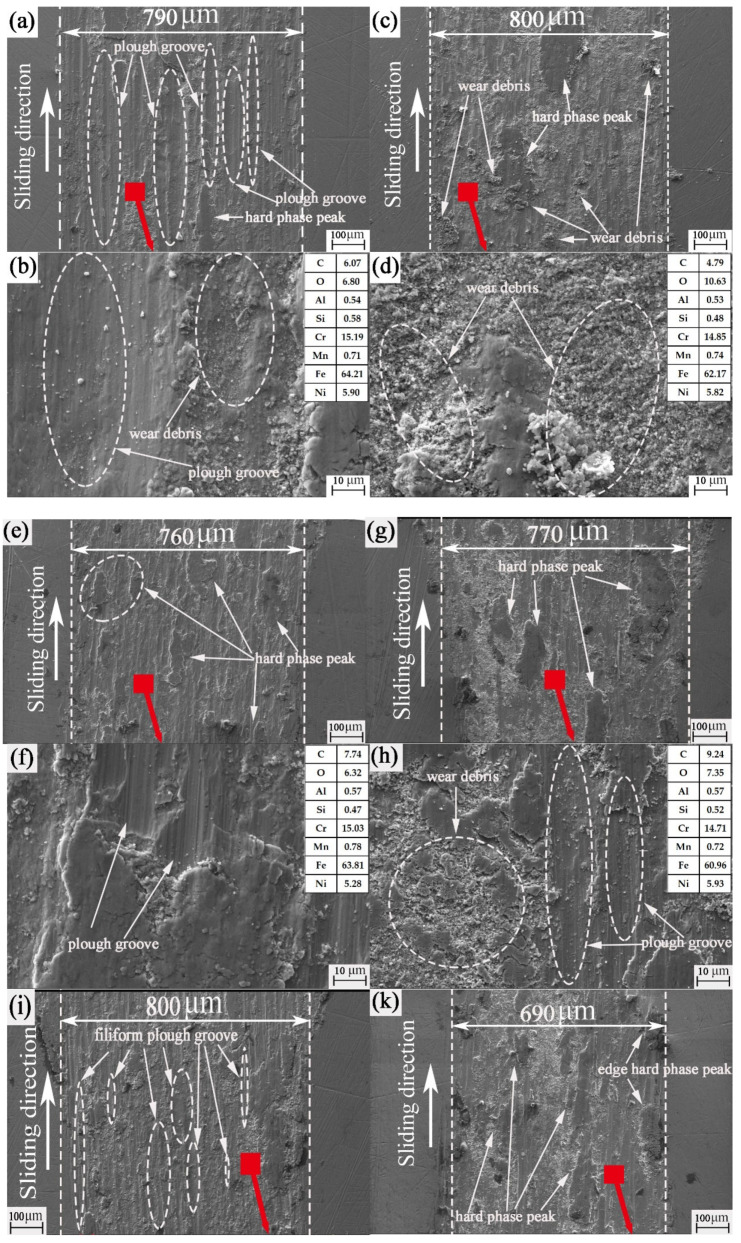

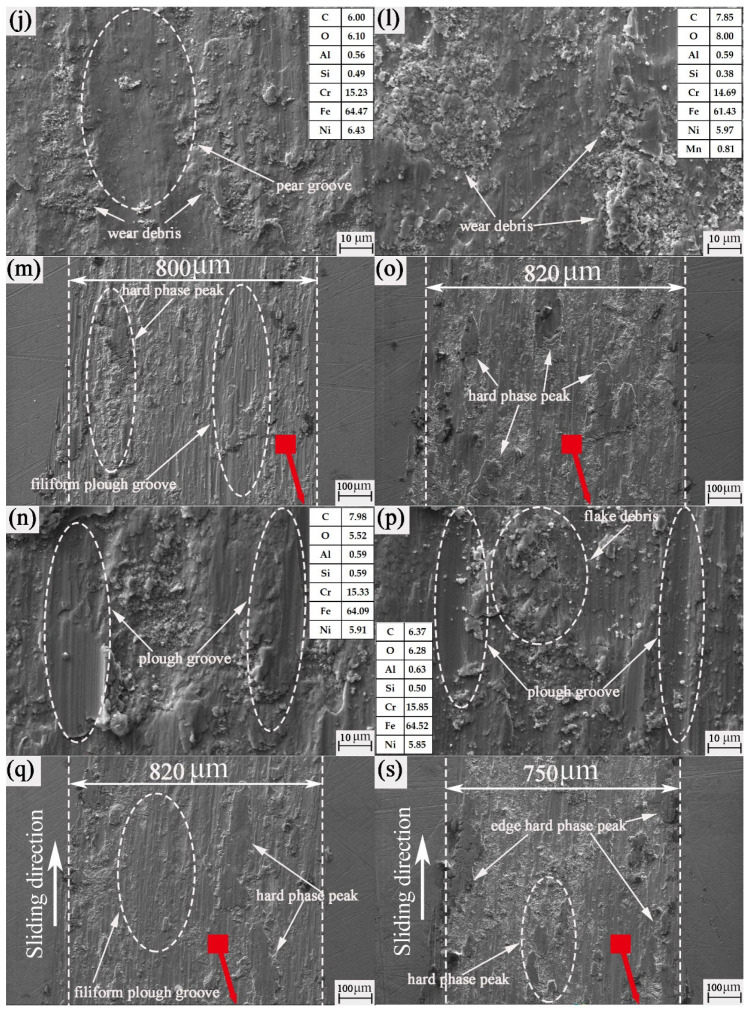

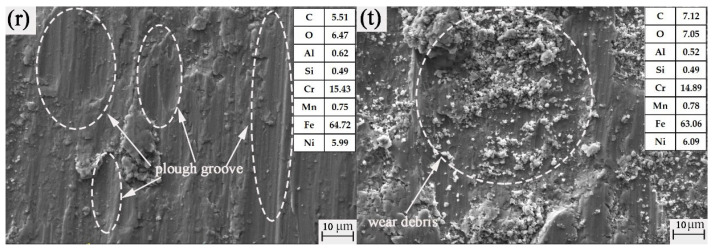
SEM images of the untextured worn plates: (**a**,**b**) untextured rotation radius of 15 mm; (**c**,**d**) untextured rotation radius of 22.5 mm; (**e**,**f**) 0.4 mm groove width mechanical texture rotation radius of 15 mm; (**g**,**h**) 0.4 mm groove width mechanical texture rotation radius of 22.5 mm; (**i**,**j**) 0.6 mm groove width mechanical texture rotation radius of 15 mm; (**k**,**l**) 0.6 mm groove width mechanical texture rotation radius of 22.5 mm; (**m**,**n**) 0.8 mm groove width mechanical texture rotation radius of 15 mm; (**o**,**p**) 0.8 mm groove width mechanical texture rotation radius of 22.5 mm; (**q**,**r**) 1.0 mm groove width mechanical texture rotation radius of 15 mm; (**s**,**t**) 1.0 mm groove width mechanical texture rotation radius of 22.5 mm.

From Figure 9a–d, it can be seen that there are a large number of filiform plough grooves and large plough grooves in the area without texture wear marks. In addition, the hard phase peak is mainly the hard phase peak of the wear mark. Moreover, it is obvious from Figure 8d that there is a large amount of large-scale debris accumulation in and near the plough groove. This wear debris proves our inference well. That is, the wear debris on the untextured surface cannot be removed in time, and it easily accumulates inside the wear mark to form a hard phase peak of the wear mark. This is consistent with the previous analysis. It can be seen from Figure 9e–t that most of the wear mark surfaces are dominated by edge hard phase peaks. There is little wear debris in the wear-mark area, and the size of the wear debris is also small and uniform. This is particularly evident in Figure 9t. It may be that the wear debris in the middle part of the wear mark falls into the groove during the friction process of the textured surface, thus greatly reducing the wear debris at the wear mark. However, the wear debris at the edge of the wear mark did not migrate further with friction. The hard phase peak at the edge of the wear mark is formed when the wear debris not captured by the texture remains at the edge of the wear mark. Combining Table 4 and Figure 9k, we find that when the wear-mark hard phase peak and the edge hard phase peak exist at the same time, it is not necessarily true that the hard phase peak plays an important role. When the size and number of hard phase peaks in the wear mark are larger than those in the edge, the reverse effect of hard phase peaks in the wear mark will be greater than the positive effect of hard phase peaks in the edge.

According to the analysis in Figure 9 and Table 3, the chemical composition of the ball and disc are quite similar. 0Cr17Ni7Al is higher than 9Cr18 in the contents of Ni and Al only. Therefore, we can focus on the Ni and Al elements. It can be seen from the energy spectrum elements in Figure 9 that all the energy spectra contain oxygen elements. It is proved that adhesive wear and abrasive wear exist in the friction process. Under the action of friction heat and air, there is also oxidation corrosion wear. It can be seen that the process of this experiment is accompanied by multiple forms of wear. However, whether the wear debris on the ball surface has transferred to the disc surface cannot be determined in this energy spectrum element.

**Figure 10:**
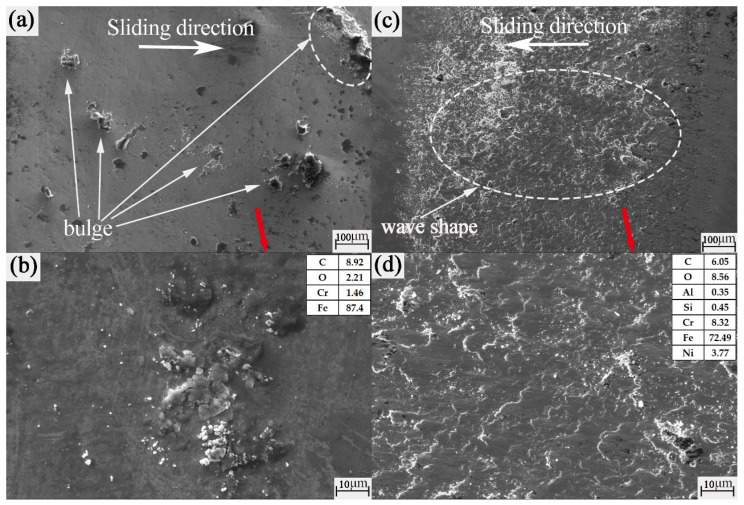
SEM images of the worn balls: (**a**,**b**) untextured rotation radius of 15 mm; (**c**,**d**) 0.8 mm groove width mechanical texture rotation radius of 15 mm.

Since the surface of the ball after texture-free friction shows a circular wear mark, the morphology is basically consistent. Therefore, we randomly select the untextured grinding balls with a radius of 15 mm as the research objects. After texture friction, the surface shape of the ball is basically quadrilateral. Therefore, the grinding ball with an 0.8 mm texture and 15 mm rotation radius is selected randomly as the research object. It can be seen from Figure 10a,b that there are sharp, hard protrusions on the surface of the wear mark. Combined with the SEM of Figure 9, it can be mutually confirmed that the interaction between the disc and the ball is a plough groove. The wear marks of the balls in Figure 10c,d are quadrilateral. This is mainly due to the friction and collision between the ball and the groove during the wear process of the first friction contact stage. The edge of the groove is equivalent to a sharp blade that cuts the grinding mark of the ball into a straight line and then becomes a quadrilateral. In Figure 10d, the abrasion marks appear as a wavy ripple, which may be caused by the impact and gluing of wear debris on the wear mark surface of the ball under the combined action of pressure, speed in the direction of motion, and friction heat. Such a wave ripple is conducive to reducing friction on the friction pair surface and plays a role in reducing friction.

Combining Table 3 and Figure 10, we can see that there is no obvious distinction between the energy spectrum elements of untextured discs and balls. It is not easy for us to judge whether the material of the ball has transferred to the disc; but from the energy spectrum elements of the ball in Figure 10d, we can see the appearance of the Al element. In addition, the mass fraction of the Ni element has obviously increased. It can be boldly inferred that the texture of the disc material was transferred to the ball. 

**Figure 11:**
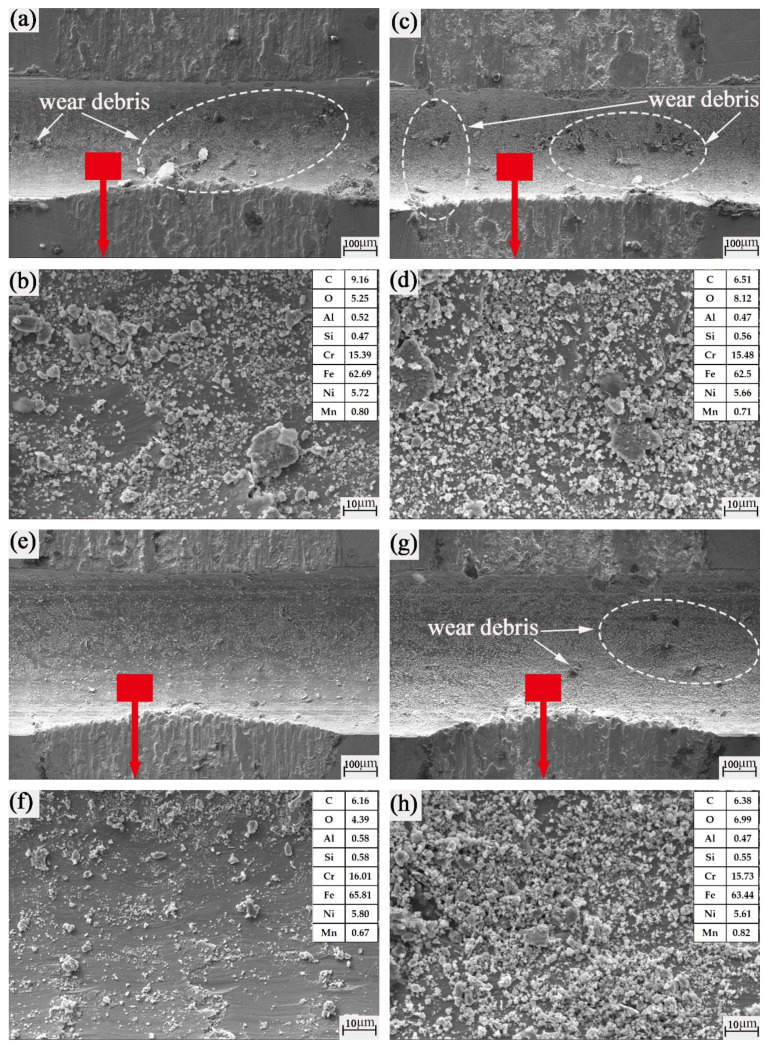

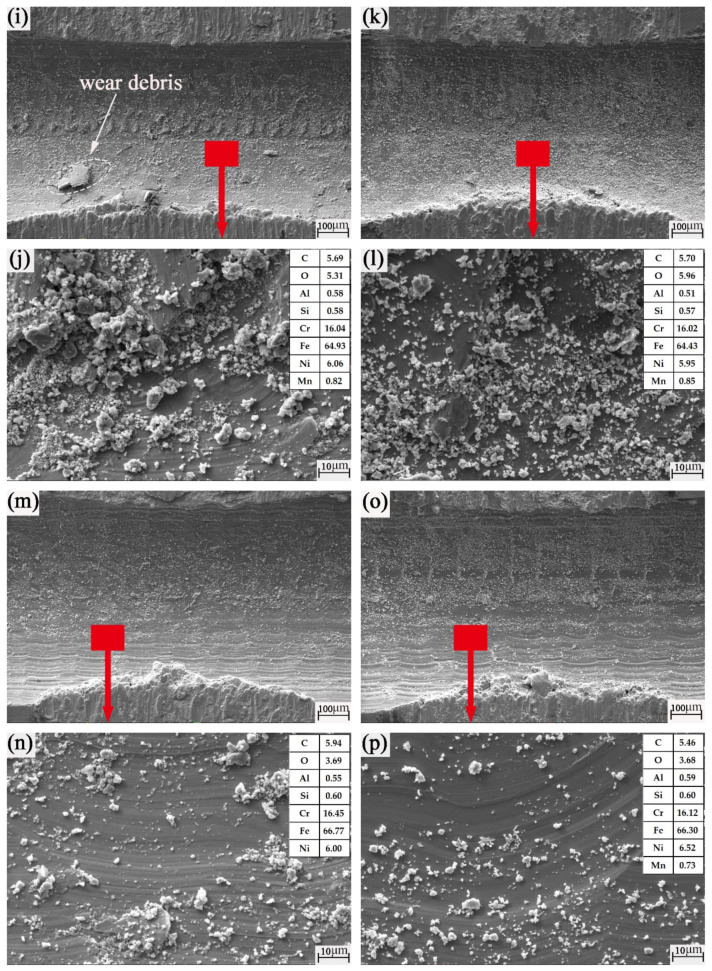
SEM images of the groove textured worn plates: (**a**,**b**) 0.4 mm groove width mechanical texture rotation radius of 15 mm; (**c**,**d**) 0.4 mm groove width mechanical texture rotation radius of 22.5 mm; (**e**,**f**) 0.6 mm groove width mechanical texture rotation radius of 15 mm; (**g**,**h**) 0.6 mm groove width mechanical texture rotation radius of 22.5 mm; (**i**,**j**) 0.8 mm groove width mechanical texture rotation radius of 15 mm; (**k**,**l**) 0.8 mm groove width mechanical texture rotation radius of 22.5 mm; (**m**,**n**) 1.0 mm groove width mechanical texture rotation radius of 15 mm; (**o**,**p**) 1.0 mm groove width mechanical texture rotation radius of 22.5 mm.

It is found from the analysis in Figure 11 that the groove bottom of the micromachined texture is very smooth and flat. As shown in Figure 11a,c,e,g,i,k,m,o, the groove edge is obviously marked by the impact and cutting of the ball and groove. This further confirms our judgment that the quadrilateral wear marks of the grooved texture ball are caused by the cutting of the ball by the edge of the groove. In addition, it is found in Figure 11a,c,g,i that the large-sized wear debris falls into the groove. This shows that the wear debris is well captured and stored by the groove. However, it can be seen from Figure 11b,d,f,h,j,l,n,p that a large amount of fine wear debris is stored in the groove. That is to say, the main form of wear is abrasive wear. Combined with the change of the oxygen element in Table 3 and Figure 11, it is proved again that abrasive wear, adhesive wear and oxidation wear occur simultaneously in the friction process. Therefore, the final wear results are integrated and coupled by a variety of wear forms.

## 5. Conclusions

In this paper, the influence mechanism of different groove widths, friction impacts and climbing heights on the friction and wear properties of micromechanical groove texture on the surface of 0Cr17Ni7Al stainless steel was studied at the nano-fractal scale. The two main results are summarized as follows:

(1) Both macro contacts and micro contacts conform to Hertz theory. From the perspective of nanoscale fractals, the surface asperities after multiple magnifications of the fractal are similar. The whole friction process can be divided into two friction contact stages. At the initial stage of friction, the micro-convex body makes contact, and the initial micro-wear debris is formed along with friction. The ball collides with the disc and generates a climbing effect. At this time, it is in the first friction contact stage. The friction collision between the ball and the disc produces collision vibration, which causes the wear debris to roll and slip. A hard phase peak is formed under the combined action of friction collision, friction heat generation and rapid cooling. When the ball is ground to a flat surface, the contact between friction pairs is mainly plane-to-plane. At this point, it is in the second friction contact stage. At this stage, the hard phase peak directly impacts the effect of friction reduction and wear resistance. The wear-mark hard phase summit increases friction and wear. The edge hard phase peak will increase the effect of friction reduction and wear resistance.

(2) Groove texture can be prepared using micromachining. The grooves in groove texture have a valuable function of storing wear debris. When the friction radius is 15 mm, the friction coefficient of all grooved textures is obviously lower than that of untextured surfaces, and the antifriction performance is better. However, the wear rate of 0.4 mm texture and 0.6 mm texture is higher than that of untextured surfaces. This shows that not all groove textures are superior to untextured surfaces. When the friction radius is 22.5 mm, the wear rate of all textures is obviously lower than that of untextured surfaces. However, as concerns the friction coefficient, the friction coefficient of the 1.0 mm groove texture is slightly higher than that of untextured surfaces. Obviously, not all textures have better antifriction performance. This shows that the choice of groove width has a great influence on the tribological properties of groove texture. In all groups of experiments, the lowest average friction coefficient of 0.8 mm texture with a friction radius of 15 mm is σ = 0.745. At this time, the total number of collisions is 24,000, and the total climbing height is 408 mm. The lowest wear rate of 1.0 mm texture with a friction radius of 22.5 mm is ω = 3.118 × $10^{-4}mm^{3}/N\cdot mm$. At this time, the total number of collisions reached 24,000, and the total climbing height was 499.2 mm. However, the maximum friction coefficient reached is σ = 0.898. The friction reduction and wear resistance of the final groove texture are the result of the coupling of the surface morphology, contact mode, friction and collision, vibration and rolling, climbing height and friction heat of friction pairs. 

Suggestions for future work include:

1. Calculating and analyzing friction and wear with the aid of artificial intelligence technology;
2. Promoting surface texture technology to other widely used materials;
3. Comprehensively analyzing the influence of multi-factor coupling such as speed, load and friction heat on friction reduction;
4. The team will also conduct in-depth research on friction and wear prediction of material surfaces in the future.

## Figures and Tables

**Table 1:** Dimensional parameters of the specimens.

	Material	Hardness	Surface Roughness	Number ofGrooves (pcs.)	Groove Width (mm)	Groove Depth (mm)	Groove Length (mm)
Ball	9Cr18	64HRC	0.014 µm	0	0	0	0
untextured	0Cr17Ni7Al	42HRC	0.05 µm	0	0	0	0
0.4 mm texture	0Cr17Ni7Al	42HRC	0.05 µm	60	0.4	0.15	12
0.6 mm texture	0Cr17Ni7Al	42HRC	0.05 µm	40	0.6	0.15	12
0.8 mm texture	0Cr17Ni7Al	42HRC	0.05 µm	30	0.8	0.15	12
1.0 mm texture	0Cr17Ni7Al	42HRC	0.05 µm	24	1.0	0.15	12

**Table 2:** Main parameters of micromachining equipment.

	Value	Unit
Spindle speed	13000	r/m (s)
Feed speed	1000	mm/m (F)
Slotting speed	100	%
Cutting speed	100	%
Cutting angle Maximum depth of each layer	0.02 0.009	mm mm

**Table 3:** Chemical composition and mass fraction of friction pair.

	Material	S	P	Al	C	Mn	Si	Ni	Cr
Plate	0Cr17Ni7Al	0.03	0.04	0.75~1.5	0.09	1.0	1.0	6.5~7.75	16~18
Ball	9Cr18	0.03	0.04	-	0.9~1.0	0.8	0.8	0.06	17~19

**Table 4:** Experimental conditions for rotation test.

	Test Radius (mm)	Rotation Speed (r/min)	Load (N)	Time (min)	Average Friction Coefficient	Wear Rate $\left(10^{-4}mm^{3}/N\cdot mm\right)$
Untextured	15	60	10	20	0.895	5.219
	22.5	40	10	20	0.867	5.140
0.4 mm texture	15 22.5	60 40	10 10	20 20	0.765 0.772	6.330 3.208
0.6 mm texture	15 22.5	60 40	10 10	20 20	0.764 0.779	5.993 3.155
0.8 mm texture	15 22.5	60 40	10 10	20 20	0.785 0.745	4.778 3.894
1.0 mm texture	15	60	10	20	0.779	4.279
	22.5	40	10	20	0.898	3.118

**Table 5:** Collision parameters with different rotation radii.

	Number of Grooves (PCs.)	Test Radius (mm)	Climbing Height per Circle (mm)	Total Friction Turns	Total Climbing Height (mm)	Total Number of Collisions (times)
Untextured	0	15	0.000	1200	0	0
	0	22.5	0.000	800	0	0
0.4 mm texture	60 60	15 22.5	0.004 0.004	1200 800	288 192	72,000 48,000
0.6 mm texture	40 40	15 22.5	0.009 0.009	1200 800	432 288	48,000 32,000
0.8 mm texture	30 30	15 22.5	0.017 0.017	1200 800	612 408	36,000 24,000
1.0 mm texture	24	15	0.026	1200	748.8	28,800
	24	22.5	0.026	800	499.2	19,200

**Table 6:** Maximum wear depth of disc test piece at 15 mm.

Untextured	0.4 mm Texture	0.6 mm Texture	0.8 mm Texture	1.0 mm Texture
20 μm	34 μm	24 μm	19 μm	21 μm

**Table 7:** Maximum wear depth of disc test piece at 22.5 mm.

Untextured	0.4 mm Texture	0.6 mm Texture	0.8 mm Texture	1.0 mm Texture
28 μm	19 μm	19 μm	25 μm	18 μm

## Data Availability

The data is available on reasonable request from the corresponding author.

## References

[1] Kumar M., Kim D. (2010). Static Performance of Hydrostatic Air Bump Foil Bearing. Tribol. Int..

[2] Zhou Q., Hou Y., Chen C. (2009). Dynamic stability experiments of compliant foil thrust bearing with viscoelastic support. Tribol. Int..

[3] Valizadeh Y.Y., Ahmadi N., Abbaspour-sani E. (2017). A thermal-calorimetric gas flow meter with improved isolating feature. Microsyst. Technol..

[4] Choudhury R., Das U.J., Ceruti A., Piancastelli L., Frizziero L., Zanuccoli G., Daidzic N.E., Rocchi I., Casano G., Piva S. (2013). Visco-elastic effects on the three dimensional hydrodynamic flow past a vertical porous plate. Int. Inf. Eng. Technol. Assoc..

[5] Guo J., Peng R., Du H., Shen Y., Li Y., Li J., Dong G. (2020). The Application of Nano-MoS2 Quantum Dots as Liquid Lubricant Additive for Tribological Behavior Improvement. Nanomaterials.

[6] Zhao J., He Y., Wang Y., Wang W., Yan L., Luo J. (2016). An investigation on the tribological properties of multilayer graphene and MoS 2 nanosheets as additives used in hydraulic applications. Tribol. Int..

[7] Zhang X., Luster B., Church A., Muratore C., Voevodin A.A., Kohli P., Aouadi S., Talapatra S. (2009). Carbon nanotube—MoS2 composites as solid lubricants. ACS Appl. Mater. Interfaces.

[8] Rabaso P., Ville F., Dassenoy F., Diaby M., Afanasiev P., Cavoret J., Vacher B., Le Mogne T. (2014). Boundary lubrication: Influence of the size and structure of inorganic fullerene-like MoS 2 nanoparticles on friction and wear reduction. Wear.

[9] Pershin V., Ovchinnikov K., Alsilo A., Stolyarov R., Memetov N. (2018). Development of Environmentally Safe Lubricants Modified by Grapheme. Nanotechnol. Russ..

[10] Nagarajan T., Khalid M., Sridewi N., Jagadish P., Walvekar R. (2022). Microwave Synthesis of Molybdenum Disulfide Nanoparticles Using Response Surface Methodology for Tribological Application. Nanomaterials.

[11] Kumar V.G.B., Pramod R., Reddy H.K.R., Ramu P., Kumar K.B., Madhukar P., Chavali M., Mohammad F., Khiste S.K. (2021). Investigation of the Tribological Characteristics of Aluminum 6061-Reinforced Titanium Carbide Metal Matrix Composites. Nanomaterials.

[12] Lu C., Shi P., Yang J., Jia J., Xie E., Sun Y. (2020). Effects of surface texturing on the tribological behaviors of PEO/PTFE coating on aluminum alloy for heavy-load and long-performance applications. J. Mater. Res. Technol..

[13] Arenas M.A., Ahuir-Torres J.I., García I., Carvajal H. (2018). Tribological behaviour of laser textured Ti6Al4V alloy coated with MoS2 and graphene. Tribol. Int..

[14] Xiong D.S., Qin Y.K., Li J.L., Wan Y., Tyagi R. (2015). Tribological properties of PTFE/laser surface textured stainless steel under starved oil lubrication. Tribol. Int..

[15] Li J., Liu S., Yu A., Xiang S. (2018). Effect of laser surface texture on CuSn6 bronze sliding against PTFE material under dry friction. Tribol. Int..

[16] Saeidi F., Meylan B., Hoffmann P., Wasmer K. (2016). Effect of surface texturing on cast iron reciprocating against steel under starved lubrication conditions: A parametric study. Wear.

[17] Cunha A., Elie A.-M., Plawinski L., Serro A.P., Botelho R., Almeida A., Urdaci M., Durrieu M., Vilar R. (2016). Femtosecond laser surface texturing of titanium as a method to reduce the adhesion of Staphylococcus aureus and biofilm formation. Appl. Surf. Sci..

[18] Basset S., Heisbourg G., Pascale A., Benayoun S., Valette S. (2022). Effect of Texturing Environment on Wetting of Biomimetic Superhydrophobic Surfaces Designed by Femtosecond Laser Texturing. Nanomaterials.

[19] Wood M.J., Servio P., Kietzig A.-M. (2021). The Tuning of LIPSS Wettability during Laser Machining and through Post-Processing. Nanomaterials.

[20] Yu H., Huang W., Wang X. (2013). Dimple patterns design for different circumstances. Lubr. Sci..

[21] Tang W., Zhou Y., Zhu H., Yang H. (2013). The effect of surface texturing on reducing the friction and wear of steel under lubricated sliding contact. Appl. Surf. Sci..

[22] Yang L., Ma W., Gao F., Xi S. (2022). Effect of Groove Width on Micromachine Groove Texture Tribology Characteristics of 0Cr17Ni7Al. Coatings.

[23] Huang W., Wang X. (2013). Biomimetic design of elastomer surface pattern for friction control under wet conditions. Bioinspir. Biomim..

[24] Etsion I. (2005). State of the art in laser surface texturing. J. Tribol. Trans. ASME.

[25] SUN J., Huo D., LI B., Xing K., Wang W., Yang Z. (2020). Wear performance of electric pruning scissors based on bionic Micro-structure. J. Agric. Mach..

[26] Chen S., Qian G., Yang L. (2019). Precise control of surface texture on carbon film by ion etching through filter: Optimization of texture size for improving tribological behavior. Surf. Coat. Technol..

[27] Ahn S., Park H., Cho J., Park C., Park J., Lee H., Hong K., Bong S., Yi J. (2021). Reactive-ion-etched glass surface with 2D periodic surface texture for application in solar cells. Optik.

[28] Natsu W., Ikeda T., Kunieda M. (2007). Generating complicated surface with electrolyte jet machining. Precis. Eng..

[29] Kern P., Veh J., Michler J. (2007). New developments in through-mask electrochemical micromachining of titanium. J. Micromech. Microeng..

[30] Zhang P., Jia R., Tao K., Jiang S., Dai X., Sun H., Jin Z., Ji Z., Liu X., Zhao C. (2019). The influence of Ag-ion concentration on the performance of mc-Si silicon solar cells textured by metal assisted chemical etching (MACE) method. Sol. Energy Mater. Sol. Cells.

[31] Zhao Y., Liu Y., Chen W., Wu J., Chen Q., Tang H., Wang Y., Du X. (2020). Regulation of surface texturization through copper-assisted chemical etching for silicon solar cells. Sol. Energy.

[32] Yang J., Shen H., Jiang Y., Sun L. (2019). Controllable fabrication and mechanism study of textured ultra-thin silicon wafers via one-step Cu-assisted chemical etching. Mat. Sci. Semicond. Proc..

[33] Yang L., Ma W., Gao F., Xi S. (2022). Effect of EDM and Femtosecond-Laser Groove-Texture Collision Frequency on Tribological Properties of 0Cr17Ni7Al Stainless Steel. Coatings.

[34] Han J., Zhang F., Van M.B., Vleugels J., Braem A., Castagne S. (2021). Laser surface texturing of zirconia-based ceramics for dental applications: A review. Mater. Sci. Eng. C Mater. Biol. Appl..

[35] Meng R., Deng J., Duan R., Liu Y., Zhang G. (2019). Modifying tribological performances of AISI 316 stainless steel surfaces by laser surface texturing and various solid lubricants. Opt. Laser Technol..

[36] Yang L., Ma W., Gao F., Xi S. (2022). Effect of Different Laser Groove Texture Collation Frequency on Tribological Properties of 0Cr17Ni7Al Stainless Steel. Materials.

[37] Ping G., Ehmann K.F. (2013). An Analysis of the Surface Generation Mechanics of the Elliptical Vibration Texturing Process. Int. J. Mach. Tools Manuf..

[38] Yang L., Ma W., Gao F., Li J., Deng M., Liu Z., Ma L., Meng H. (2021). Study on Tribological Properties of groove texture in surface micromachining. Tool Technol..

[39] Muller V.M., Yushchenko V.S., Derjaguin B.V. (1980). On the influence of molecular forces on the deformation of an elastic sphere and its sticking to a rigid plane. J. Colloid Interface Sci..

[40] Greenwood J.A., Williamson J.B.P. (1966). Contact of nominally flat surfaces. Proc. R. Soc. Lond. Ser. A Math. Phys. Sci..

[41] McCool J.I. (1986). Predicting Microfracture in Ceramics Via a Microcontact Model. J. Tribol..

[42] Chang L., Jeng Y.R. (2012). Effects of negative skewness of surface roughness on the contact and lubrication of nominally flat metallic surfaces. Proc. Inst. Mech. Eng..

[43] Pogačnik A., Kalin M. (2013). How to determine the number of asperity peaks, their radii and their heights for engineering surfaces: A critical appraisal. Wear.

[44] Majumdar A., Bhushan B. (1990). Role of Fractal Geometry in Roughness Characterization and Contact Mechanics of Surfaces. J. Tribol..

